# Digital detection of dementia (D^3^): a study protocol for a pragmatic cluster-randomized trial examining the application of patient-reported outcomes and passive clinical decision support systems

**DOI:** 10.1186/s13063-022-06809-5

**Published:** 2022-10-11

**Authors:** Michael J. Kleiman, Abbi D. Plewes, Arthur Owora, Randall W. Grout, Paul Richard Dexter, Nicole R. Fowler, James E. Galvin, Zina Ben Miled, Malaz Boustani

**Affiliations:** 1 Comprehensive Center for Brain Health, Department of Neurology, University of Miami Miller School of Medicine, 7700 W Camino Real, Suite 200, Boca Raton, FL 33433 USA; 2 Indiana University School of Medicine, Indianapolis, IN 46202 USA; 3 Center for Health Innovation and Implementation Science, Indiana Clinical and Translational Science Institute, Indianapolis, IN, 46202 USA; 4 Indiana University Bloomington School of Public Health, Bloomington, IN 47405 USA; 5 Regenstrief Institute, Inc., Indianapolis, IN 46202 USA; 6 Indiana University Center for Aging Research, Indianapolis, IN 46202 USA

**Keywords:** Alzheimer’s disease and related dementias, Electronic health records, Machine learning, Patient reported outcome, Clinical decision support

## Abstract

**Background:**

Early detection of Alzheimer’s disease and related dementias (ADRD) in a primary care setting is challenging due to time constraints and stigma. The implementation of scalable, sustainable, and patient-driven processes may improve early detection of ADRD; however, there are competing approaches; information may be obtained either directly from a patient (e.g., through a questionnaire) or passively using electronic health record (EHR) data. In this study, we aim to identify the benefit of a combined approach using a pragmatic cluster-randomized clinical trial.

**Methods:**

We have developed a Passive Digital Marker (PDM), based on machine learning algorithms applied to EHR data, and paired it with a patient-reported outcome (the Quick Dementia Rating Scale or QDRS) to rapidly share an identified risk of impairment to a patient’s physician. Clinics in both south Florida and Indiana will be randomly assigned to one of three study arms: 1200 patients in each of the two populations will be administered either the PDM, the PDM with the QDRS, or neither, for a total of 7200 patients across all clinics and populations. Both incidence of ADRD diagnosis and acceptance into ADRD diagnostic work-up regimens is hypothesized to increase when patients are administered both the PDM and QDRS. Physicians performing the work-up regimens will be blind to the study arm of the patient.

**Discussion:**

This study aims to test the accuracy and effectiveness of the two scalable approaches (PDM and QDRS) for the early detection of ADRD among older adults attending primary care practices. The data obtained in this study may lead to national early detection and management program for ADRD as an efficient and beneficial method of reducing the current and future burden of ADRD, as well as improving the annual rate of newly documented ADRD in primary care practices.

**Trial registration:**

ClinicalTrials.gov Identifier: NCT05231954. Registered February 9, 2022.

## Background

Alzheimer’s disease and related dementias (ADRD) affects millions of Americans, impacting both the patients themselves and their caregivers. ADRD carries with it an annual societal cost of more than $200 million; meanwhile, half of Americans living with ADRD remain undiagnosed [1–7]. If a diagnosis is made, it is usually 2 to 5 years after the onset of symptoms and when ADRD is likely in the mild to moderate stage. This delay in diagnosis drastically reduces the likelihood of improving outcomes from drug and non-drug therapeutic interventions and prolongs the expenses of medical care [6–9]. Additionally, delayed detection of ADRD increases the associated cognitive, functional, and psychological disabilities which results in a significant burden for patients, families, and the entire society [1, 10]. Previous approaches to early ADRD detection have included the use of cognitive screening tests and biological markers [2, 3, 5, 11–13]. However, few primary care systems are designed to routinely detect ADRD and when approached, as many as 38% of patients refuse cognitive screening tests in primary care [14–21]. Additionally, most tests that are available to primary care fail to detect ADRD in individuals with high cognitive capacity and are rarely appropriate for multi-cultural populations who speak languages other than English and who may have lower educational attainment or quality of education [13].

In order to address these barriers to detection of ADRD in primary care, we developed a machine learning (ML) algorithm that can predict ADRD both 1 and 3 years prior to detection by using routine electronic health record (EHR) data, the Passive Digital Marker (PDM) [22]. The algorithm was trained using structured and unstructured data from three EHR datasets: diagnosis (Dx), prescriptions (Rx), and medical notes (Nx). Individual algorithms derived from each of the three datasets were developed and compared to a combined one that included all three datasets. These machine learning algorithms were trained and tested by using EHR data of incident ADRD cases and non-ADRD controls. The variables of the algorithm include demographic features like age, sex, and race as well as medical features extracted from the Rx, Dx, and Nx categories of the health record. The PDM leverages widely available electronic health record (EHR) data and advances in machine learning algorithms to achieve 80% accuracy for one-year and 77% accuracy for 3-year prediction horizons [22].

Early ADRD detection in a healthcare delivery system including primary care is challenging; many rating scales take significant time or require specialized training and access to diagnostic services if a person is identified to be at risk [3, 5, 11–13]. To address this, several brief, culturally and linguistically sensitive patient-reported outcome tools and sustainable approaches for the early detection and staging of ADRD were developed [23–27]. Patient-reported outcome approaches can overcome barriers for early detection of ADRD in primary care practices and can monitor ongoing symptoms of ADRD as well as how these symptoms affect patient functioning. The Quick Dementia Rating Scale (QDRS) was created for observational study and was tested and validated against the Clinical Dementia Rating (CDR) Scale: a gold standard measure used in the NIA ADRCs and in many trials that use cognition, function, and behavior as outcomes. The QDRS has 85% diagnosis accuracy in comparison to the CDR [23]. The addition of the QDRS as a patient-reported outcome to the PDM in the planned trial is to enhance the approach to capture real-world patient functioning, cognition, mood, and behavior that is not routinely captured in the EHR.

An integrated approach based on the above two ADRD early detection approaches (the PDM and the QDRS) may overcome the current barriers to early ADRD detection in a cost- and time-efficient manner that can be generalized across any primary care practice. These two approaches may allow feasible and scalable segmentation of patient populations including a high-risk group that could be targeted for further invasive, expensive, and time consuming cognitive or biological screening tests.

We will conduct two pragmatic cluster-randomized trials in diverse primary care practices in Indiana and Florida to evaluate the practical utility and effect of the PDM, the QDRS, and the combined approach (PDM + QDRS) in improving the annual rate of new documented ADRD diagnosis. Our primary hypothesis is that in comparison to usual care, the combined approach (PDM + QDRS) will increase the incidence rate of ADRD over the subsequent 12 months from 6 to 13%. Our secondary hypothesis is that in comparison to usual care alone, the combined approach (PDM + QDRS) will have higher acceptance rates for recommended ADRD diagnostic work-up among patients following a positive screen from 44 to 66%.

## Materials and methods

### Setting

The two trials will be conducted in two separate healthcare systems; the first trial will be conducted at Eskenazi Health, and within 12 months a second replicated trial will be conducted at the University of Miami Health. Both trials will have identical methodology and would compare the performance of the three approaches in increasing the incidence rate of new ADRD diagnoses subsequently documented in the EHR by the primary care clinics.

Participating practice sites are diverse primary care practices in central Indiana and south Florida. This includes 10 federally qualified health centers affiliated with Eskenazi Health in Indianapolis and 10 primary care practices in South Florida affiliated with University of Miami (UHealth). Eskenazi Health a large integrated healthcare system serving underprivileged older residents of Marion County including African Americans (50%) and dual eligible Medicaid and Medicare beneficiaries with a low socioeconomic status (50%). UHealth has primary care practices serving older adults residing in Miami-Dade, Broward, and Palm Beach Counties and serving underrepresented groups of African Americans (19%) and Hispanic (45%) populations throughout South Florida.

At each of the two research sites (south Florida and Indiana), we plan to enroll approximately 3600 patient participants; thus, we expect a total of 7200 patients enrolled in the two trials. We will approach the practice manager and the physician leader of each primary care clinic to randomize the clinic into one of three early detection approaches: clinics providing usual care without PDM or QDRS, clinics who will use PDM alone, and clinics who will use both PDM and QDRS. To randomly allocate each practice to one of the three screening approaches, we will use a computer-generated randomization scheme. The allocation of each practice will be stored on a secure sever, with access restricted to only the co-PIs. The PDM and the QDRS will be embedded within the routine visit process for each clinic. Practice managers and physicians from each clinic will be aware of their assigned group following group allocation, as they will either be provided information about the PDM and/or the QDRS or informed that they will not receive recommendations from either the PDM or QDRS. We have obtained a waiver of informed consent from the local Institutional Review Board to review retrospectively (after at least two consecutive years of screening) the EHR systems of each clinic to calculate the annual rate of new ADRD diagnoses. Moreover, we will review other EHR data to measure processes of diagnostic assessment following positive screen such as referral rate for diagnostic assessments for early ADRD and patient acceptance rate of undergoing such assessments.

### Inclusion criteria

Retrospective EHR review will be performed on patients who (1) are 65 years or older, (2) have at least one visit to primary care practice within the past year, (3) are able to communicate in either English or Spanish, and (4) have available EHR data from at least the past 3 years.

### Exclusion criteria

Participants who will not be examined include those that (1) have a prior ADRD or MCI diagnosis as determined by ICD-10 code, (2) have evidence of any history of prescription of cholinesterase inhibitors or memantine, (3) have serious mental illness such as bipolar or schizophrenia as determined by ICD-10 code, and (4) are a permanent resident of a nursing facility.

### Practice context

We will interview the practice managers by implementing a one-time completion of the Practice Assessment Tool (PAT) for each clinic enrolled in the two pragmatic trials to describe the context of the healthcare systems. The Centers for Medicare and Medicaid Services developed the PAT to determine the volume-to-value transformation phase of primary care practices. The PAT uses 27 milestones to assess a practice’s transformation. Each milestone is scored on a 0–3 scale based on interviews with practice staff; not yet implementing that milestone (score 0), getting started with implementation (score 1), implementing and partially operating (score 2), or functioning and performing well (score 3). All scores milestone subscales and milestone totals are reported as a percentage (i.e., observed score divided by possible score, multiplied by 100), ranging from 0 to 100. The PAT was validated in an observational study of 622 primary care practices in five states in the Midwest and was found to be predictive for practice transition into an Alternative Payment Model.

### Study design

**Fig. 1:**
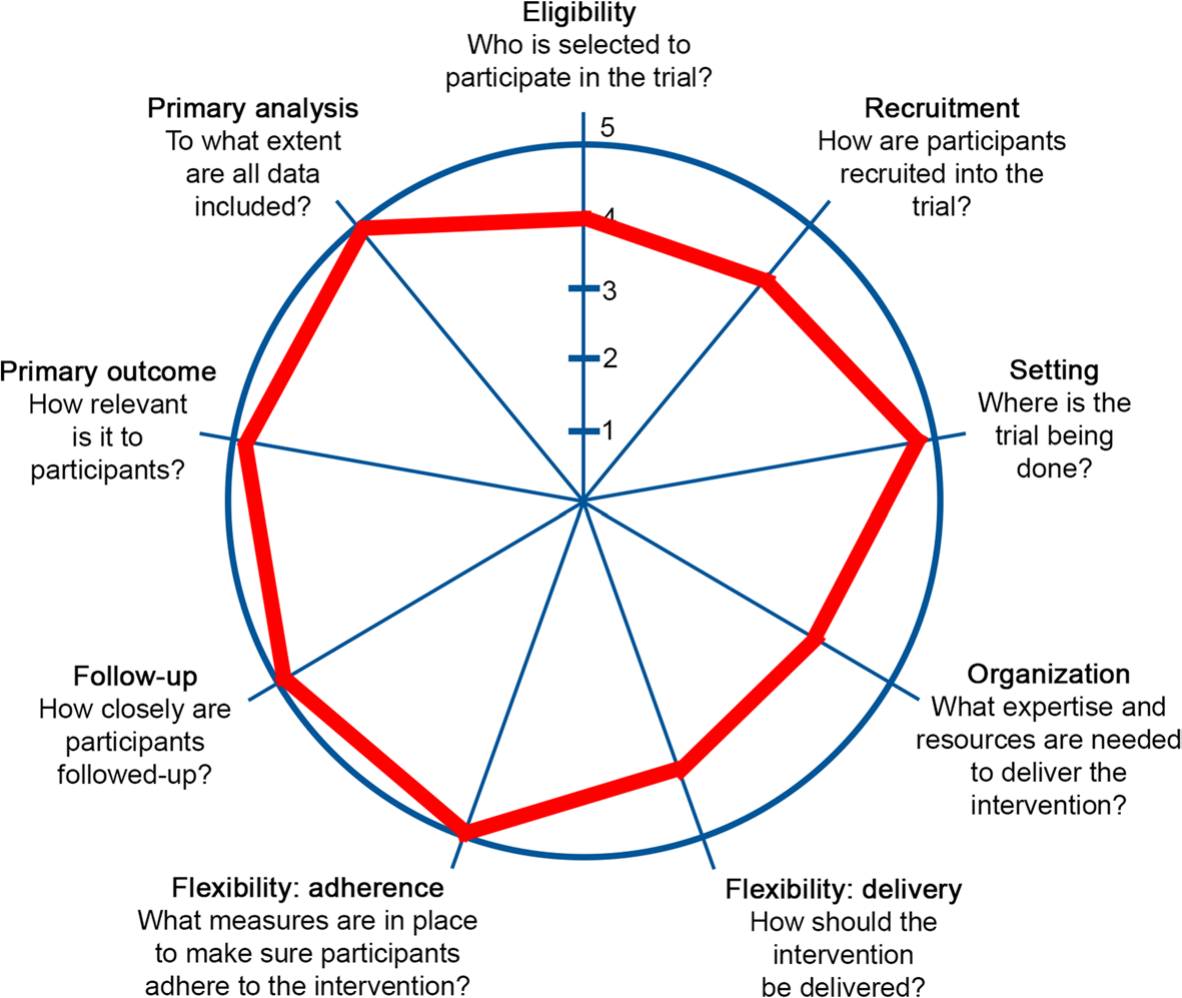
Our two trials are highly pragmatic as indicated by scores of 4 and 5 (*‘most pragmatic’*) in the PRECIS-2 summary wheel depicted here. The diverse primary care settings, minimal inclusion/exclusion criteria, highly relevant clinical outcomes to patients and providers, close EHR-based data follow-up, and expertise in developing scalable low-cost cognitive assessments results in a very pragmatic design. We have strong partnerships with local primary care clinics. Patient input was received in designing QDRS and study flow. Other pragmatic elements include not asking clinicians to deny patients any routine procedures such as cognitive tests and using a variety of patient identification methods

Our research design is predicated on the notion that patient screening would appropriately identify a more targeted and appropriate group of primary care patients for referral for diagnostic services. To achieve this, we calculate both the PDM and QDRS and embed them within the EHR systems of diverse primary care practices in Indianapolis and south Florida. We will then run the independent pragmatic cluster-randomized controlled comparative effectiveness trial of the three approaches (usual care, PDM only, PDM + QDRS), with pragmatism explored and depicted using the PRECIS-2 tool (Fig. 1). To randomly assign each practice to one of the three screening approaches, we will use a computer-generated randomization scheme. Our interdisciplinary scientific teams developed and tested both the PDM and the QDRS. We leveraged the widely available EHR data and the advantage of ML to develop the PDM with an approximate 80% accuracy for 1-year and 3-year prediction horizons [22]. Our team has also developed and tested the QDRS as a practical 2–3-min patient-reported outcome tool for both early detection and staging of ADRD [23].

Table 1 outlines the design and enrollment process for this study.

**Table 1:** Schedule of enrollment, interventions, and assessments

	**Study Period**		
	**Allocation**	**Enrollment**	**Post-allocation**
**Timepoint**	-T1	T0	T1 (12 months)
Eligibility Screen		X	
Randomization of practices	X		
**Interventions**			
Usual care		X	
PDM only		X	
PDM + QDRS		X	
**Assessments**			
Clinical evaluation and screening		X	
EHR review			X
PAT			X

*Abbreviations*: *PDM* Passive Digital Marker, *QDRS* Quick Dementia Rating Scale, *HER* Electronic Health Records, *PAT* Practice Assessment Tool

### Integration into EHR systems

We are integrating the PDM and the QDRS within the usual clinical encounter to accommodate the clinical flow, data capture, and results display needs of the primary care practices. We are leveraging the processes and technologies currently and successfully in use every day in our clinical partners’ primary care practices. Both practice sites use Epic as their EHR and its related clinical decision support (CDS) engine and patient-completed survey platforms. The EHR-based CDS system will display the results of the screening to clinicians to promote the interpretation of the results by healthcare providers and patients. Over the past 3 years, we have partnered with our Epic EHR clinical informatics colleagues to deploy various CDS tools such as patient reported surveys, predictive algorithms for social frailty and deprescribing recommendations [28–39]. These CDS tools provide clinicians with a graphical summary of the results with a focus on the factors driving these results. A similar approach has been adopted in the two trials, tailored through the extensive configuration options available in the Epic CDS engine. Patient registration at the primary care clinic will trigger the following CDS process as well as a prompt to assign the QDRS to the patient. The CDS engine will query the latest PDM ML results calculated for the patient. Screening results will be displayed within the EHR user interface, including actionable clinician-directed recommendations related to appropriate diagnostic work-up.

### Primary and secondary outcomes

The two pragmatic trials will use data captured by the EHR to assess the primary outcome measure of any new ADRD case identified (documented in the EHR) within 12 months of the visit where the screening result is displayed (index visit) [14, 40]. The secondary outcome measures will be any services related to cognitive impairment diagnostic assessment in the 12-month post visit period after the index visit when the screening result is displayed. Specifically, the metrics of diagnostic assessment will be evaluated as proportions of patients with a record of 1 or more of (a) laboratory tests for TSH, serum B_12_, folate, or syphilis; individually or combined at any point during the 90 days after index; (b) neuropsychological testing, including testing by psychologist or physician, technician administrator, computer, or other providers during the 12 months after index date; (c) brain imaging testing (computed tomography, magnetic resonance imaging, positron emission tomography, magnetic resonance angiogram) of the head and neck, brain, or skull during the 12 months after index date; and (d) medications approved for management of ADRD (cholinesterase inhibitors, memantine) during the 12 months after index date.

### Data and safety monitoring plan

We will construct a data safety monitoring plan that will be monitored by the co-PIs and a three-member Data Safety and Monitoring Board. The team will use the Epic EHR system log to monitor the uptake and usage of the deployed screening approaches of the PDM and QDRS within the Epic CDS on a quarterly basis. If data uptake in the interim appears problematic, we will be able to alter the user interface based on feedback. Risk–benefit ratio assessment will be performed on an annual basis. It is unlikely that the trial would be stopped early. The NIH will make the final decision on accepting the Data Safety and Monitoring Board’s recommendation about discontinuation of any component of the study. Database security is maintained using a multi-layered approach to both limit access and the ability to alter data.

Adverse event rates associated with screening interventions are low and are expected to vary little between the intervention and control groups in the two pragmatic trials. We will present blinded adverse event data to the co-PIs throughout the trials. We plan to present unblinded adverse events data to the Data Safety and Monitoring Board panel when requested and at the annual meetings. If there is evidence of elevated adverse events, the co-PIs will consult with the study team and use an adverse event form to report injuries or other adverse events caused by the intervention and detected through sources listed below. No serious adverse events are expected for the two pragmatic trials. Our CHOICE trial [15] found no negative impact of screening on depression, anxiety, quality of life, or health care utilization; thus, the only expected adverse event is potential loss of confidentiality.

### Steering committee

The Steering Committee for this project consists of the three principal investigators: Dr. Boustani and Dr. Ben Miled for Indiana, and Dr. Galvin for Florida. Each are responsible for translating the strategic plans of the research proposal into operational plans, policies, and procedures, as well as manuscript preparation and reporting. They oversee the deployment and analysis of the machine learning models for dementia prediction in the targeted sites of the pragmatic trial. The Steering Committee met weekly during the first 12 months of the award period, afterwards continuing to meet on a biweekly basis along with co-investigators to monitor the progress of the study objectives and disseminate completed study aims through publications and other network activity. The remaining study team members, including the research coordinators at each site, the data manager and data analysts, and the research assistants, support the study by conducting recruitment, consenting, and data collection at each site as well as data tracking in REDCap and reporting on progress.

### Analysis

In each trial, we will calculate the annual incidence rate of EHR documented ADRD across the three comparative clusters (usual care, PDM alone, PDM + QDRS) using nonlinear mixed models to account for clinic-clustered data. In the two pragmatic trials, different patients are randomized (via their clinics) to each of the 3 arms, making the outcomes independent between the three screening approaches; however, data will still be correlated due to clustering of patients within clinics, which will be handled by random effects in the mixed models. We hypothesize the incidence rate will be 6% using usual care [40] and 13% with the screening approach that combines usual care + PDM + QDRS. Incidence rates for all arms will be calculated along with their 95% confidence interval.

For our secondary hypothesis that the combined approach will increase acceptance rates of diagnostic assessment, the analyses will be the same as described for the primary hypothesis except that the outcome will be acceptance rates for recommended ADRD diagnostic work-up following positive screen. We hypothesize the acceptance rate will be 44% using usual care alone [15] and 66% with the screening approach that combines usual care, the PDM, and the QDRS.

## Discussion

### Leveraging EHR and machine learning

Growth in data captured by various EHR systems, increased access to inexpensive computational power and advancements in ML algorithms offer opportunities to develop targeted and scalable approaches for the early detection of ADRD. These approaches may allow feasible and scalable identification and segmentation of patient populations including a high-risk group that could be targeted for further in-depth cognitive or biomarker tests. However, one of the most difficult aspects of working with EHR data is its heterogeneous nature, with many different data types (e.g., continuous versus categorical, structured versus unstructured) and the common state of missing values for any number of variables per patient, as not all tests are administered to each patient or recorded correctly if they are. While such heterogeneity makes it difficult to apply various ML algorithms [41], the ubiquity of EHR data makes it vital that we aim to leverage this resource for purposes of identifying early ADRD.

Relatively few research studies have explored the use of both the EHR data and ML algorithms to detect ADRD [42–45]. Two studies developed electronic search algorithms for identifying ADRD from EHR notes [43, 44]. Another study examined the impact of combining natural language processing with the presence of ADRD-related diagnosis codes and ADRD medications [42]. A fourth study used Bayesian network to develop an algorithm that reached 80% accuracy related to the diagnosis of ADRD based on five cognitive exams that are not available at scale in most EHR systems used in primary care practices [45]. While the accuracy from using Bayesian networks is good, a significant amount of neuropsychological data, computation, feature engineering, and expert-guided bootstrapping is required in order to use this type of ML algorithm. The use of ML algorithms that require capturing detailed neuropsychological or invasive biological data from large numbers of older adults in primary care practice is simply not scalable nor sustainable. While the few studies that used ML algorithms and existing data in the EHR demonstrate utility in identifying patients diagnosed with more advanced ADRD, they do not support the early screening of patients at risk for developing ADRD. Thus, balancing the capability of the most accurate ML algorithms, the available data within the current EHR systems and the focus on ADRD screening is the essential core of developing and implementing a low-cost scalable early detection approach for ADRD in primary care practices. At Indiana University, the PDM for early detection of ADRD has demonstrated 80% accuracy for one-year and 77% for three-year prediction horizons [22].

### Using patient-reported outcomes to improve early detection of ADRD

Previous approaches to early ADRD detection have included the use of cognitive screening tests, in-depth neuropsychological testing, or biomarkers [2, 3, 5, 11–13]. Using biomarkers for early detection of ADRD is not scalable in primary care due to their invasive nature (blood or lumbar puncture), their cost (MRI, PET), and their accessibility (rural or underserved areas). Patient-reported outcome approaches can overcome these barriers for early detection of ADRD in primary care practices and can monitor ongoing symptoms of ADRD as well as how these symptoms affect patient functioning. They can create efficient and cost-effective clinical encounters with providers while also empowering patients and family caregivers to engage in early detection of ADRD [13, 24, 46].

The Quick Dementia Rating Scale (QDRS) has been shown to be effective as both tool for early detection and staging for ADRD with 85% accuracy and is highly correlated with the Clinical Dementia Rating scale, neuropsychological testing, and ADRD biomarkers (MRI, CSF, PET) [23, 27]. Completion of the QDRS can offer several advantages above and beyond what is captured through EHR review including (a) capture of non-memory symptoms (e.g., orientation, problem-solving, daily functioning) that are both disturbing to patients and families and are more likely to be accepted as a change that requires medical attention; (b) provide information about the patient’s real-world functioning; (c) provide information at visits for new patients where prior EHR data may not be available; (d) capture of progression over time; and (e) allow for staging of ADRD in a brief, valid, and time- and cost-effective manner [13, 23, 27].

### Limitations

We may encounter barriers such as stakeholder buy-in, unintended consequences, and alert fatigue. We will overcome these barriers by (1) working closely with the leadership of the primary care practices to ensure stakeholder buy in, (2) leveraging the trusted relationship between the patient and her primary care team, (3) fully integrating the proposed PDM and QDRS within the clinical flow of the primary care practices, and (4) working with the Epic Team to trouble shoot EHR integration barriers and minimize alert fatigue. Based on our extensive preliminary studies, the combined approach of using the PDM and the QDRS would outperform the current usual care or the use of the PDM alone.

## Conclusions

Our two trials will inform the scientific community and the health care system about the performance of two scalable approaches in early detection of ADRD and improving the annual rate of new documented ADRD in primary care practices. These trials will provide some knowledge about the specific characteristics of the primary care patients, and their attitudes toward early detection of ADRD and subsequent referral for appropriate diagnostic and management services.

### Trial status

This protocol is version number 1.1, dated August 10, 2022. Recruitment began on July 5, 2022, and is anticipated to conclude on February 1, 2025.

## Data Availability

De-identified data will be shared with the community via the Open Source Framework at the conclusion of the study. It will also be replicated in the NIA IMPACT Collaboratory. The datasets analyzed during the current study and statistical code are available from the corresponding author on reasonable request, as is the full protocol.

## Abbreviations

ADRD: Alzheimer’s disease and related disorders
CDR: Clinical Dementia Rating
CDS: Clinical decision support
Dx: Diagnosis
EHR: Electronic health record
MCI: Mild cognitive impairment
ML: Machine learning
Nx: Medical notes
PAT: Practice Assessment Tool
PDM: Passive Digital Marker
QDRS: Quick Dementia Rating Scale
Rx: Prescription
